# Dynamics of infestation of tracers lambs by gastrointestinal helminths under a traditional management system in the North of Tunisia

**DOI:** 10.1051/parasite/2012194407

**Published:** 2012-11-15

**Authors:** H. Akkari, M. Gharbi, M.A. Darghouth

**Affiliations:** 1 Laboratoire de Parasitologie, École Nationale de Médecine Vétérinaire 2020 Sidi Thabet , Université de La Manouba Tunisie

**Keywords:** helminths, sheep, epidemiology, Tunisia, helminthes, mouton, épidémiologie, Tunisie

## Abstract

The authors present a survey of gastrointestinal helminths of sheep on permanent pastures in the extreme north region of Tunisia (Mediterranean climate). Dynamic infestation of animals has been monitored by using batches of three tracer lambs introduced each two months during 2004 and 2005. These lambs were kept in the pens of veterinary school of Sidi Thabet (Tunisia) during three months and then necropsied. Faecal and blood samples were took from tracer lambs each two months during the whole period, and from animal flock only during 2004. The main helminth genera encountered were *Trichostrongylus* spp., *Teladorsagia* spp., *Strongyloides papillosus* and Anoplocephalidea; occasionaly were found *Nematodirus*, *Oesophagostomum*, *Chabertia*, *Cooperia*, *Trichuris* and *Paramphistomum*. The egg count of the ewes and lambs in the flock showed two peaks. For both ewes and lambs there is a gradual increase from January with a peak in May-June. This first peak is considered to be due to acquisition of infective larvae during the rainy and cold season, as evident from the worm burdens of tracer lambs. The second peak was exclusively observed in ewes during late autumn-early winter (November-December); it has two origins: infestation by third larvae stage and the periparturient rise. The worm burdens of tracer lambs showed that there was a gradual accumulation of nematodes from September- October, reaching a peak in March-April; a very low or naught infection is reported during the dry period (July-August). Infection by Anoplocephalidea was higher during the dry season. This study is primordial for a comprehensive control programme implementation against gastrointestinal helminths.

## Abstract (fr)

Les auteurs présentent une étude quantitative et qualitative des helminthes gastro-intestinaux des ovins dans le nord de la Tunisie (climat méditerranéen). La dynamique d’infestation des animaux a été contrôlée en utilisant des lots de trois agneaux traceurs introduits tous les deux mois durant une période qui s’est étalée sur deux années, 2004 et 2005. Après leur retrait du pâturage, les agneaux traceurs ont été gardés à l’École vétérinaire de Sidi Thabet (Tunisie) durant trois mois, puis autopsiés. Des échantillons de matière fécale et de sang ont été pris sur des agneaux traceurs à chaque période de pâture et aussi sur des animaux d’un troupeau de la région d’étude durant l’année 2004. Les principaux helminthes rencontrés étaient *Trichostrongylus* spp., *Teladorsagia* spp., *Strongyloides papillosus* et Anoplocephalidea, et occasionnellement ont été trouvés *Nematodirus*, *Oesophagostomum*, *Chabertia*, *Cooperia*, *Trichuris* et *Paramphistomum*. Le décompte des oeufs par gramme chez les brebis et les agneaux dans le troupeau a montré deux pics. Pour les deux types d’animaux, il y a une augmentation graduelle à partir de janvier, avec un pic en mai-juin. Ce premier pic est considéré comme lié à l’acquisition des larves infestantes durant la saison pluvieuse et froide de l’année, comme en témoigne la charge parasitaire des agneaux traceurs. Le second pic a été exclusivement observé chez les brebis en fin d’automne-début d’hiver (novembre-décembre) ; il a deux origines : l’infestation par les L3 automnales et l’augmentation du nombre d’oeufs de nématodes émis par les femelles autour de la mise bas. La charge parasitaire chez les agneaux traceurs a montré qu’il y avait une accumulation progressive des nématodes à partir de septembre-octobre, atteignant un maximum en mars-avril, alors qu’une infestation très faible ou nulle est signalée durant la période sèche de l’année (juillet-août). L’infestation par des Anoplocephalidea était plus élevée pendant la saison sèche. Cette étude était nécessaire pour la mise en oeuvre d’un programme de contrôle efficace des helminthes gastro-intestinaux.

## Introduction

Small ruminant industry plays a significant role in food security and as an income source for many resource-poor families in most developing countries. In Tunisia, sheep rearing has a high social and financial importance, for instance it is contributing to 50 % of red meat national production (Rekik & Ben Hammouda, 2000). There are many constraints to small ruminant industry development including a range of health problems, insufficient nutrition and inadequate management practices. In the humid and sub-humid regions, gastrointestinal parasites, and particularly nematode infestation, represent one of the main constraint to production of small ruminants (Mage *et al.*, 1998). Parasitic infections affect small ruminants’ productivity with decreased growth rates and reproductive performances, high mortality rates, bad quality products leading to a dramatic increased of production costs (Waller, 1997; Perry & Randolph, 1999). The control of these infestations needs an excellent knowledge of their epidemiological features and requires important and persistent expenses (Michel, 1976, 1985; Armour, 1980). In the North of Tunisia, anthelmintics are still used by the majority of traditional flock owners for treating clinical cases of infestation. That is why we emphasise the need for relevant epidemiological data to design and implement rational preventive programmes against sheep helminthiasis in the studied region.

The importance of knowledge of the seasonal population trends and availability of exogenous stages of sheep helminths for design control programmes has been well documented in other countries (Armour, 1980; Brunsdon, 1980; Katerina *et al.*, 2009). Indeed, each stage of the gastrointestinal exogenic phase is conditioned by climatic factors, specially temperature and humidity which determine both activity and viability of exogenous stages conditioning the epidemiological feature of gastrointestinal helminthiases.

Due to the high correlation between tracer lambs’ helminths burdens and pastures contamination by free parasites stages (Muller, 1968; Anderson, 1972; Bailey *et al.*, 2009), the use of worm-free lambs as tracer animals is a valuable epidemiological tool to monitor free stages infestation of the pastures (Southcott *et al.*, 1976).

A study of the dynamics of infestation of tracer lambs by *Fasciola hepatica* was carried out during two successive years (Akkari *et al.*, 2011). As these tracer lambs were subject to an helminthological necropsy covering the totality of the gastrointestinal tract, the present paper reports the results of this study for gastrointestinal nematodes and discuss their application for prevention of these parasitic infestation under the usual management practices of the North West of Tunisia.

## Material and Methods

### Study Region

The present survey was carried out in a northwestern Tunisian region (Sejnane) located in the Governorate of Bizerte (37° 06’ N; 09° 10’ W); it is a swampy region characterized by an hydromorphic and clayey soil, an humid Mediterranean climate with a mean annual rainfall of 1,000 mm.

### Animals

The animal material of the study was exhaustively described in the work of Akkari *et al.* (2011) focused on *F. hepatica*. This study has been carried out on an extensively managed flock of Tunisian Barbarine sheep breed, which was retained for antecedents of clinical cases of gastrointestinal parasites. The whole flock consist of 154 animals; we randomised 15 lambs (four to eight months age) and 15 ewes (three to six years old); it grazes in scrub and shoal all over the year. The choice of the flock was conditioned by the owner’s good willingness to cooperate and by a history of gastrointestinal helminthiasis clinical cases (diarrhoea, cachexia, growth rate decrease, and mortality).

 The animals are exclusively fed on the natural resources of the region, they browse on shrubs and trees (kermes oak, oleaster, mastic...) of the Mediterranean bush and graze on Gramineae and Leguminosae of natural pastures and fallow lands (uncultivated lands during one or more years) and stubble. During cold season, the animals are under a rudimentary shelter and outdoor when it is warmer. The ewes lamb twice a year: the main during the autumn and the second is smaller in spring (Rekik *et al.*, 2000).

 Self-medication with albendazole generics (Anthelben S, Medivet) was used by the owner only on sheep with clinical symptoms suggesting gastrointestinal helminthiasis without any knowledge of the drug activity, the doses and with no distinction between adults and lambs. In order not to interfere with the dynamics of transmission, the farmer was asked not to change any breeding management on his animals. During the survey, 36 tracer Barbarine four to six months old male lambs of 14.8 kg (+/- 3.6) average weight were sequentially introduced in the flock. Before being introduced, each tracer animal was housed for one month at the pen of veterinary school (Sidi Thabet, Tunisa). They were vaccinated against enterotoxaemia (Coglavax, CEVA Santé Animale), treated against scabies with diazinon (Néocidol 250, Medivet) and drenched with albendazole (Anthelben S, Medivet) at the conventional dose of 7.5 mg/kg twice at two days interval. The absence of any gastrointestinal and lung helminths was verified by coproscopic examination. The tracer lambs were introduced in the monitored flock by groups of three animals for a period of two months, then transferred to the Veterinary School of Sidi Thabet (Tunisia), where they were kept in pens for three months. At the end of this period, lambs were slaughtered for an helminthological autopsy.

Faecal and blood samples were realised every two months during exclusively 2004 on the monitored flock. Faecal egg counts (FEC) were estimated using a modified McMaster technique (Whitlock, 1948). Blood samples were collected for haemoglobinaemia estimation. The presence of anaemia was defined on the basis of threshold of 9 g. dl-1 (Blood & Radostitis, 1989).

Monitoring And Autopsy of the Tracer Lambs

As described in the paper on fasciolasis (Akkari *et al.*, 2011), the tracer lambs were monitored for FEC and haemoglobin (Hb) at the end of the housing period of three months at the Veterinary School of Sidi Thabet. Thereafter, the tracer lambs were necropsied by method as described by Sissay *et al.* (2007), with except that no digestions of abomasal mucosa were carried out. At necropsy the abomasum, small and large intestine were separated, opened, and washed in warm water. The total contents and washings of the abomasum and small intestines were separately collected and made up to a total volume of 2 l water. The total content of caecum and large intestine is washed through 500 μm and 1000 μm sieves one placed on the other and visually inspected for large bowel nematode.

Duplicate samples of aliquot size 1:10 (200 ml) were taken from the washings of each of the abomasum and small intestine contents, following thorough stirring. Worms were distinguished as adult male and female nematodes, and were preserved in 5 % formalin solution. In addition, the presence of adult tapeworms in the small intestine was established by visual inspection; rumens were opened and inspected for the presence of rumen flukes (paramphistomes), and the livers were sliced open to search for the presence of liver flukes. Identification of species was based on the morphological characteristics described in Soulsby (1982) and Euzeby (1969). Cestodes identification concerned exclusively most prevalent species.

### Climatic Data

**Fig. 1.**
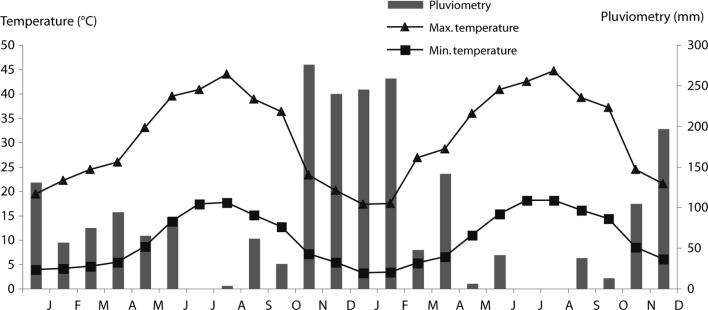
Mean monthly minimum and maximum air temperature for the period of study.

Mean, minimal, maximal temperature and rainfall for 2003, 2004 and 2005, were gathered free of charge from the meteorological station of Sejnane (Courtesy of “Institut National de la Météorologie”, Tunisia) (Fig. 1).

### Statistical Analysis

The number of helminths recovered from the tracer lambs were expressed relatively to the grazing corresponding period. In order to compare variables between different groups, the comparison of different means was realised using the analysis of variance test (ANOVA) (Schwartz, 1993). The presence of correlation between the number of gastrointestinal nematodes in tracer animals, the FEC and haemoglobinaemia, was assessed by regression analysis. Statistical analyses were performed with SPSS 10© for Windows© software with a cut off probability of 0.05 (Schwartz, 1993).

## Results

### Gastrointestinal Helminths Recovery At Necropsy (Table I)

**Fig. 2.**
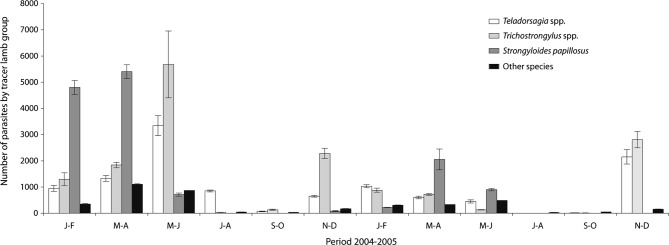
Bimonthly evolution of different gastrointestinal nematodes. Other species: *Haemonchus contortus*, *Nematodirus* spp., *Cooperia oncophora*, *Trichuris* spp., *Chabertia ovina*, *Oesophagostomum venulosum*

Roughly half: 45.5 % and 52.8 % of the parasitic population of tracer lambs were found in the abomasum and small intestine respectively, while in the large intestine the number of parasites recovered has not exceeded 1.7 % (Fig. 2). The total worm count (TWC) decreased or vanishes during the dry seasons and increased during the wet seasons of each year. There was a significant difference in the TWC during the two years of study, we noted 32,020 and 13,343 worms (70.5 % and 29.5 %) in 2004 and 2005 respectively (p = 0.03). In addition, TWC peaked in late spring-early summer 2004 (May-June) and in winter 2005 (November-December) (Fig. 2).

**Table I.** Proportion (in %) of different nematode genus recovered after necropsy of tracer lambs

Nematode species	Prevalence (%)	Rate of population (%)	Range of infection intensity
*Trichuris* spp.	100.00	1.32	1 – 44
*Teladorsagia* spp.	86.10	25.22	10 – 1680
*Trichostrongylus axei*	77.80	18.43	20 – 1270
*Trichostrongylus* spp.	77.80	16.42	40 – 4440
*Strongyloides papillosus*	58.35	31.18	10 – 2200
*Nematodirus* spp.	52.78	5.20	10 – 610
*Chabertia ovina*	41.70	0.22	1 – 19
*Oesophagostomum venulosum*	38.90	0.16	1 – 37
*Haemonchus contortus*	36.15	1.83	1 – 210
*Cooperia oncophora*	8.30	0.06	10

The most frequent nematodes found in the digestive tract of tracer lambs (representing 91.25 % of the total worm burden) were *Teladorsagia* spp. (*T. circumcincta*, 94 %; *T. trifurcata*, 4.5 %; *T. lyrata*, 1.5 %); *Trichostrongylus* spp. (*T. axei*, 52 %; *T. colubriformis*, 45.5 %; *T. vitrinus*, 2.5 %) and *Strongyloides papillosus.*

Infestation with *Teladorsagia* spp. and *Trichostrongylus* spp. increased in November-December, with a peak during grazing period (May-June 2004) or winter (January-February, 2005); subsequently the acquisition of new infections falling in summer-early autumn (September-October) or even earlier (July 2005).

Infestation of tracer lambs by *S. papillosus* begun in November-December, increased in winter, with a peak in spring (March-April); the infestation was annihilated from July to August. The parasite infestation intensity by *S. papillosus* in 2005 was lower than 2004 (reduction of 77 %).

*Nematodirus* spp. (*N. spathiger*, 85 %; *N. filicollis*, 9.5 %; *N. battus*, 5.5 %) were poorly represented in the digestive tract, like other nematodes it appeared in the autumn, the infestation increased in winter and reached its peak in spring.

*Trichuris* spp. (*T. ovis*, 78 %; *T. globulosa*, 22 %) infested all tracer lambs, it was present during all over the year with a peak in spring (March-April), but the intensity remained very low compared to other nematodes.

*Haemonchus contortus*, *Chabertia ovina*, *Oesophagostomum venulosum* and *Cooperia oncophora* species were the lowest represented in sheep digestive tract.

**Fig. 3.**
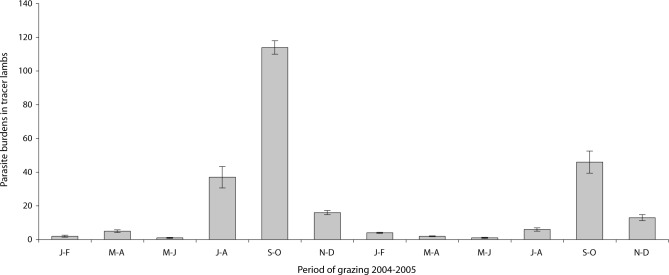
Bimonthly evolution of anoplocephalidae collected from small intestines of tracer lambs.

The infestation prevalence by tapeworms in the gut reached 75.5 %, consisting of five species of anoplocephalidae: *Moniezia expansa*, *M. benedeni*, *Stilesia globipunctata*, *Thysaniezia ovilla* and *Avitellina centripunctata*. Infestation by these tapeworms was inverted in comparison to nematodes infestation dynamics. Indeed, during 2004 and 2005 the number of anoplocephalidae increased in summer (July-August) with a peak in autumn (September-October); it decreased in winter and spring. The number of anoplocephalidae in tracer lambs was higher in 2004 (175, 70 %) than 2005 (72, 30 %) (Fig. 3).

Only four specimens of *Paramphistomum* spp. were found in the rumen of one tracer lamb during November-December 2004.

### Nematode Faecal Egg Counts

The variation of the mean FEC in tracers lambs, showed a significant positive correlation with adult worms burdens (coefficient of correlation r = 0.623; p < 0.001). In the flock, the mean overall prevalence of gastrointestinal nematodes was 93 % and 93 % respectively in ewes and lambs (Table II).

**Table II.** Variation of prevalence, coproscopy and haemoglobnemia in the flock at each grazing period during 2004.

	Ewes			Lambs		
Year period	Prevalence (%)	FEC (egg/gram)	Hb (g/100 ml)	Prevalence (%)	FEC (egg/gram)	Hb (g/100 ml)
Jun.–Feb.	84.6	282.69 (0–1800)	9.15	86.7	199.33 (0–2000)	9.72
Mar.–Ap.	93.3	296.33 (0–750)	8.52	100.0	152.85 (0–750)	11.85
May.–June	100.0	777.33 (15–2615)	7.71	86.7	415.66 (0–1400)	8.65
July–August	93.3	369.66 (0–1515)	7.09	93.3	499.66 (0–2515)	6.74
Sept.–Oct.	84.6	136.92 (0–300)	9.78	93.3	174.33 (0–400)	9.80
Nov.–Dec.	100.0	1855.33 (200–5400)	8.97	100.0	423 (15–1500)	10.74
Mean	92.63	619.71	8.53	93.33	310.805	9.58

FEC: faecal egg count; Hb: haemoglobin.

The infestation prevalence in the flock was high during all periods of the year (between 78 and 100 %). During 2004, average FEC was higher in ewes (620) than lambs (311). The faecal egg counts exhibited two peaks: a first during late spring (May-June) for ewes with an average maximum of 777.5, and in July-August for lambs with an average maximum of 500; a second peak was observed in November-December with an average maximum FEC of 1,855 and 423 in ewes and lambs respectively.

### Heamoglobinaemia

There was no statistically significant correlation between gastrointestinal nematodes and hemoglobin values in tracer lambs (coefficient of correlation r = 0.06; p = 0.73). In the flock, mean haemoglobinaemia varied between 8.53 and 9.58 g/dl in ewes and lambs respectively. The difference between mean haemoglobinaemia of ewes and lambs was statistically significant (p = 0.003). In addition, there was a significant correlation between haemoglobinaemia and FEC for only ewes (r = 0.24; p = 0.03). During 2004, haemoglobinaemia were higher in March-April (8.52 and 11.85 g/dl in ewes and lambs, respectively) and lower during July-August (7.09 and 6.74 g/dl in ewes and lambs, respectively) (Table II).

## Discussion

In northern Tunisia, particularly in the Sejnane region, climate is favourable for sheep infestation by gastrointestinal nematodes, and tracer lambs revealed very high infestation prevalences (reaching 100 %). Climatic factors, mainly temperature and rainfall are limiting factors for the development and survival of infective nematode gastrointestinal larvae (Levine, 1963), influencing then the epidemiology of nematodes infestation. With the onset of rains in autumn, the parasitic burdens increased gradually, reaching a peak in spring. The lack of rain after June explains the very low nematode rate infection in tracer lambs grazing during July-August (Fig. 2). Temperature may play a role in the peak infestation by *Teladorsagia* spp. and *Trichostrongylus* spp. during May-June 2004 and November-December 2005, and for *S. papillosus* in March-April.

The nematode fauna found in the present study was similar to that reported in France (Cabaret, 2002), Spain (Uriarte *et al.*, 2003) and Rabat region (Morocco) (Pandey *et al.*, 1990). The low large intestine parasites burdens were similar to those reported in Middle Atlas and Rabat regions (Cabaret, 1983; Pandey *et al.*, 1990). The major difference with these Moroccan studies is the absence of *Chabertia* spp. and the occurrence of *Trichuris* spp. infection all over the year.

The difference of species number between the two years was associated with important fluctuation of rainfall. Indeed, the low proportion of parasites collected in 2005, compared to 2004, was associated with high torrential rain in November and February. Consequently, in addition to the leaching of the pastures, we have recorded an extension of shoals, and then animals were constrained to pasture in higher lands with lower infestation risks. These results agree with others (Thomas, 1974; Waller & Thomas, 1978), who suggested that variations from year to year could be correlated with local climatic conditions. In addition, in 2005, due to an increase of fasciolosis incidence, the farmer increased the use of albendazole (Akkari *et al.*, 2011).

The coproscopic survey during 2004 in the flock showed very high infestation rates in both ewes and lambs (92 % and 93 %, respectively). These values are comparable with those reported in Northwest Spain (87.9 %) (Martinez-Gonziilez *et al.*, 1998), but higher than those of Pallargues (2007) in Morocco (60 %). The high values of ewes’ FEC compared to lambs can be explained by a decrease of ewes’ immunity (Kerboeuf, 1978; Herd *et al.*, 1983; Gibbs, 1986; Jansen, 1987; Mage, 1998). Indeed, in the study region, the lambing season spread throughout all the 2004, ewes are either pregnant or lactating. This immunodepression is aggravated by malnutrition since all the animals pasture through over the year, but are never supplemented. Several studies showed the crucial role of malnutrition and other parasites (such as *F. hepatica*) in increasing the negative effect of digestive parasitism (Coop & Holmes, 1996; Mejjati & Sykes, 1996; Coop & Kyriaszakis, 1999). This was shown in the present study by the low values of haemoglobinaemia in ewes compared to lambs (8.53 and 9.58 g/dl, respectively; p = 0,003), with the presence of significant correlation between haemoglobinaemia and FEC for only ewes (r of Pearson = 0.24; p = 0.03).

Due to dramatic increase of fasciolosis incidence during 2005, the farmer intensively used albendazole. We decided not to consider the flock dynamic infestation during 2005 (Akkari *et al.*, 2011). The egg count of the ewes and lambs in the flock showed during 2004 a seasonal pattern (Table II). There was a gradual increase from January with a peak in May-June. This first peak was due to acquisition of infective larvae during the rainy and cold season, as showed by the worm burdens of tracer lambs. In fact, during the January-April, major species (*Teladorsagia* spp.; *Trichostrongylus* spp.; *Nematodirus* spp.) have high capacities of development and resistance, as mentioned by others (Boag & Thomas, 1975; Mallet & Kerboeuf, 1986; Pandey *et al.*, 1993). In addition, O’Connor *et al.* (2006) reported that these nematodes are the dominant parasites in temperate areas where the cooler temperature create optimal conditions for larval development. This biotic capacity increases the pastures charge of third stage, causing a continuous infestation in this period. During late winter, the climatic conditions become suitable for an evolution of eggs rejected by ewes, leading to a dramatic increase of third stage population in pastures, coinciding with the appearance of a high number of grazing lambs. These lambs recycle the third stage form eggs eliminated by ewes, and become by their self very important egg disseminators, leading to a spring third stage peak, as shown by tracer lambs. This peak was the cause of high disease risk in all age categories. The second peak was exclusively observed in ewes during November-December; it could be explained by: (i) acquisition of an increased number of third stage in autumn as revealed by tracer lambs; (ii) the peak coincided with the lambing season (from November to January); during this period the prolificity of the nematodes increases; this phenomenon is known as periparturient rise (Kerboeuf, 1978; Gibbs, 1986; Jansen, 1987; Mage, 1998).

As shown in other regions of the world, such infections of lambs and ewes at pasture arise from two sources of infective larvae: the first from the previous autumn (so-called overwintering third instars), and the second from the eggs deposited in spring (Thomas & Boag, 1972; Donald & Waller, 1973; Uriarte & Valderrçbano, 1989). The second generation has been considered to be the main source of parasitic disease in lambs (Gibson & Everett, 1972), while the overwintering larvae are often a minor source of direct lamb infection in areas where climatic conditions delay the start of the grazing season. However, under Mediterranean conditions, this study showed that the number of parasites harvested by tracer lambs during the colder months (November-February) was 40 %. This suggests that the population of overwintering larvae could have important consequences in the performance of lambs under grazing systems in the north of Tunisia.

Tracer lambs showed that the infection rate of the flock was low of nought during the hot season (July- August); this had two origins: (i) very low or nought rainfall (July-August: 3.4 mm and 0 mm in 2004 and 2005, respectively), the pastures were unfavourable for exogenic stages development (Levine, 1963); (ii) during this period, animals grazed in dry pastures, which became sterile due to the destruction of all the exogenic stages by high temperature associated to low hygrometry, as well studied under Mediterranean climate in Australia (Anderson, 1972, 1973; Calinan 1978, 1979), in Morocco (Pandey, 1980; 1990; Ouhelli *et al.*, 1981; Cabaret, 1984), and in South Spain (Carmona, 1985). The possibility of low infestation rates by *Teladorsagia* spp., *Trichuris* spp. and *Nematodirus* spp. were associated either to low rainfall or to topographic characteristics of this region with bush, low relief, Springs, which can retain humidity allowing the survive of a residual population of nematodes or to the resistance of certain species to dryness (Mauléon & Gruner, 1984). When the climatic factors were unfavourable, development of several species were inhibited in sheep at the fourth larvae stage as reported in Morocco (Pandey *et al.*, 1980; Cabaret, 1984). No abomasal digestion were performed since the tracer lambs were kept for three months in the pens of the veterinary school of Sidi Thabet (Tunisia), and any hypobiotic larvae should be reactivated at the end of this period.

Haematological data in tracer lambs revealed the absence of correlation between nematodes infestation and haemoglobinaemia (r = 0.06; p = 0.73). This can be explained by the low infestation intensity by the haematophagous species *H. contortus* (Hoste *et al.*, 1997) (Table I), which is the main causative agent of gastroenteritis in tropical regions (Woolaston & Baker, 1996; Sissay *et al.*, 2007).

*F. hepatica* was the major aetiology of anaemia in the study region (Akkari *et al.*, 2010), with a strong correlation between haemoglobinaemia values and fluke burdens in tracer lambs (r = 0.761; p < 0.001). Most commonly prevalent species of sheep intestinal tapeworms causing heavy economic losses have been reported to be *M. expansa*, *M. benedeni* and *A. centripunctata* (El-Mukdal, 1977; Al-Khafaji & Rhaymah, 1993; Celep *et al.*, 1995). The relative ability of their intermediate host (oribatid mites) to survive on pasture at different periods of the year is relevant for the successful formulation of a control programme, as it will determine how long a pasture can remain at risk following high levels of mite biomass.

Tracer lambs showed high infestation rates reaching 75.5 %; they begin to be infested in summer with a maximum in autumn (September-October) and declines rapidly in winter and spring. Infestation occurred during hot and moderately wet periods of the year. Indeed, during July-October 2004 and 2005, we have collected 203 tapeworms, representing 82 % of the total parasites burdens. High infestation rates during autumn were due to suitable climatic factors in the Sejnane region for the development of the oribatid mites (rainfall < 50 mm; Temperature < 30 °C). Dryness and high temperatures destroy oribatid mites (Chermette *et al.*, 2003). In Rabat region (Morocco), the use of tracer lambs showed that infestation by tapeworms occurred all along the year with a peak during late spring-early summer (Pandey *et al.*, 1990). Also in Morocco, Ouhelli *et al.* (1981) showed that infestation peak occurs in spring. In Chad, Grabber *et al.* (1974) showed that infestation by *Moniezia* spp. occurs mainly during the hot and dry season.

The presence of *Paramphistomum* spp. is explained by the presence of its intermediate host in the study area. Indeed, hydrological and topographic characteristics of this region are particularly favourable for the development of the amphibious gastropod *Galba truncatula* (Jemli *et al.*, 1991).

The infestation prevalence of this fluke in tracer lambs was 2.8 % (1/36), associated with high rainfall (537 mm in November-December 2004). This result agree with those of Díaz *et al.* (2007) in Spain, who reported that the high risk periods for infection by *Paramphistomum* spp. are those following high rainfall. It is therefore possible that the importance of this fluke will continue to increase, as reported in several works carried out in Europe (Mage *et al.*, 2002; Otranto & Traversa, 2002).

The curative administration of anthelmintics performed by the sheep owner over 2004 was not effective in controlling gastrointestinal helminths in the herd. This demonstrates the necessity for a comprehensive control programme against these important parasitic diseases. The control programme in the studied region consists of two strategic anthelmintic treatments. The first, in early summer (June), when the animals are removed to the stubble, will lead to the rupture of the cycle, since the animals are free of parasites and put in free pastures. The second treatment is indicated in winter (December), targeting late pregnant ewes. The aim of this treatment is to reduce periparturient rise phenomenon, leading to a dramatic decrease of pastures’ contamination then a decrease of spring contamination. A tactic treatment in spring (March) is indicated in the studied region, targeting both lambs’ and pastures’ parasitism. However, such use of anthelmintics two to three times a year should be accompanied by practices aimed at reducing the risk of drug-resistant emergence.
